# Recovery of gait after ankle fracture surgery following early mobilization and weight-bearing

**DOI:** 10.1007/s00402-026-06472-4

**Published:** 2026-08-10

**Authors:** Leandra Bauer, Anne Sophie Schlegel, Anne Scheuer, Wolfgang Böcker, Hans Polzer, Sebastian Felix Baumbach

**Affiliations:** 1 Experimentel Orthopaedics, University Hospital Jena, Campus Eisenberg, Waldkliniken Eisenberg, Friedrich-Schiller-Universität Jena, Jena, Germany; 2 Department of Orthopaedics and Trauma Surgery, Musculoskeletal University Center Munich (MUM), University Hospital Munich, Ludwig-Maximilians-Universität München, Munich, Germany

**Keywords:** Ankle fracture, Weight-bearing, Gait analysis, Rapid recovery

## Abstract

**Background:**

Ankle fractures are among the most common fractures in adults. Despite significant advancements in surgical techniques, the postoperative treatment regimens remain conservative. This study aims to objectively evaluate gait recovery following early mobilization and weight-bearing after surgical ankle fracture treatment using instrumented gait analysis.

**Methods:**

This prospective, single-armed, longitudinal study enrolled adult patients following surgical treatment for isolated ankle fractures with early pain dependent weightbearing. Gait analysis, utilizing a treadmill integrated with a pressure plate and IMU-based motion capture systems, was conducted at 6 weeks, 3-, 6-, and 12-months post-surgery, assessing spatio-temporal and kinematic parameters. Results were compared longitudinally and against a healthy control group.

**Results:**

Longitudinal data were available for 44 patients. Significant (*p*<.001) improvements of self-paced walking velocity (SPV) were observed from 6 weeks (2.2 ± 1.1 km/h), to 3 (2.9 ± 0.9 km/h) and 6 months (3.4 ± 0.9 km/h). The spatio-temporal parameters showed significant changes between 6 weeks and all further time points only at SPV. Kinematic parameters showed significant longitudinal improvements at SPV for hip, knee, and ankle motion (including all three ankle planes). Patient gait parameters at 6 and 12 months were comparable to healthy controls, except for persistent limitations in ankle sagittal kinematics.

**Conclusion:**

Patients undergoing surgical ankle fracture repair with early mobilization and full weight-bearing showed substantial gait recovery within 6 weeks. By 12 weeks, patient gait closely resembled healthy controls, highlighting the efficacy of a pain-adapted postoperative regimen for rapid functional recovery and return to previous activity levels.

**Level of evidence:**

Level II.

## Introduction

Ankle fractures are among the most common fractures in adults. For Germany, the annual incidence has been reported to be 74 ± 32 per 100,000 individuals [1]. Ankle fractures refer to fractures to the lateral malleolus (distal fibula), medial malleolus (distal, medial tibia), and/or posterior malleolus (posterior tibia). The most common fractures are isolated fractures to the lateral malleolus. Whereas fracture incidences decrease with the number of malleoli fractured, i.e. uni- > bi- > tri-malleolar ankle fractures, the severity of the injury increases [2].

Surgical treatment strategies have evolved remarkably over the past 70 years. Especially the development of angular-stable implants and open reduction and internal fixation of fractures to the posterior malleolus have further increased the primary stability of the osteosynthesis. The percentage of angular-stable plates for lateral malleolus fractures in Germany has increased from 2% in 2005 to 34% in 2019 [1].

Paradoxically, the postoperative treatment regime has not changed. Postoperative treatment regimens comprise of two major concepts, immobilization/mobilization and non-weight-bearing/weight-bearing. The most used postoperative treatment regime is immobilization (boot or cast) and 20 kg partial weight-bearing for 6 to 8 weeks [3]. But recent studies have shown that neither early mobilization nor early weight-bearing increase the complication rate in surgically treated ankle fractures [4–7]. Controversially, early mobilization and weight-bearing rather improve the short-term outcomes (≤ 6 months) for the OMAS, range of motion (RoM), and return to work (RTW) [4].

Still these outcome parameters are limited. Patient reported outcome measures, such as the OMAS, have a ceiling-effect and a subjective bias. RoM measurements are static and subject to a possible assessment bias. The instrumented gait analysis is a dynamic and objective alternative to assess the outcome of surgery to the lower limb. Various studies already have used instrumented gait analysis to assess the outcome following surgical treated ankle fractures [8]. Still, these studies are limited as they either compared only two time points, or a single time point to a healthy reference population. Still, to better understand the rehabilitation process, measurements should be taken at multiple timepoints (assessment longitudinal progression) and be compared to a healthy reference population (assessment final outcome).

The aim of this prospective study was to assess the gait parameter progression over time and their comparison to a healthy cohort in patients treated surgically for an ankle fracture.

## Materials and methods

**Fig. 1:**
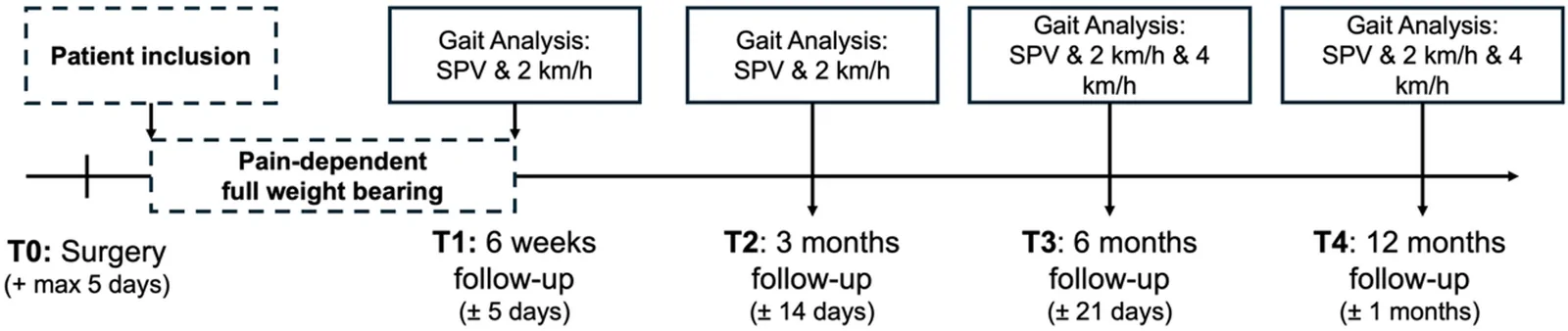
Overview of patient inclusion, surgery, and follow-up assessments. Gait analyses were performed at 6 weeks, 3 months, 6 months, and 12 months after surgery, with self-paced velocity and fixed-speed walking trials depending on the respective time point

The herein presented study is a single-armed, prospective study. The study was approved by the Institutional Review Board of the local Faculty of Medicine (IRB no. 21–0698) and part of a multicenter clinical trial (NCT05419154, registered on 2022-07-06) [9] in accordance with the Declaration of Helsinki. All participants signed informed consent. The study was conducted at a tertiary care orthopaedic trauma centre in Germany. Patients were recruited between 05/2022 and 08/2024. The general study timeline is highlighted in Fig. 1.

### Patient recruitment

Eligible were adult patients (18 ≤ age < 75 years) treated surgically for an acute, isolated ankle fracture without syndesmosis stabilization by means of syndesmotic screw(s) or a suture-button system. Syndesmotic stabilization by ventral bracing augmentation was no exclusion criteria. Ventral augmentation was performed by a FiberTape (Fa. Arthrex) shuttled through the plate and fixed to the Tubercule de Chaput (4.75 mm BioComposit SwiveLock anchor; Fa. Arthex) [10]. Study inclusion was conducted following surgical treatment. If eligible, patients were informed about the study and invited to participate.

### Surgical treatment

The surgical treatment must have followed the AO principles. At the authors’ center, the surgical treatment of ankle fractures is standardized per the following sequence: Arthroscopy, open reduction and internal fixation (ORIF) posterior malleolus, ORIF lateral malleolus, ORIF medial malleolus, syndesmotic stabilization, deltoid repair. Arthroscopy was performed for bi-/tri-malleolar ankle fractures, and any dislocated (≥ 2 mm) posterior malleolus fracture of sufficient size was addressed. Sufficient size was defined as a posterior malleolus fragment big enough to fit a 3.5 mm screw. In case of a 2-ligament injury of the syndesmotic complex (AiTFL + IOL), an augmentation of the AiTFL (Fibretape + Swift Lock Anker, Fa. Arthrex) was performed [10]. A 2-ligament injury was verified by direct visualization, arthroscopy and/or external rotation stress test under fluoroscopy (widening MCS). In case the surgeon did decide for another syndesmotic stabilization procedure (suture button system or screw), the patient was not eligible.

### Intervention

If eligible, patients were allowed to conduct pain-dependent full weight-bearing without immobilization, starting at the day of study inclusion. They were advised to follow the PRICE regime for the first two postoperative weeks to assure wound healing. After the initial two-week period, following removal of the stiches, the patients were motivated to conduct full-weightbearing as tolerated by pain. The ankle joint was not immobilize at any time-point [9].

### Instrumented gait analysis

An instrumented gait analysis was performed 6 weeks, 3 months, 6 months and 12 months post-surgery. The gait analysis was performed on a treadmill (pluto^®^ med, h/p/cosmos^®^ sports & medical GmbH, Nussdorf-Traunstein, Germany) with an integrated pressure plate (Zebris Medical GmbH, Isny, Germany). The kinematic measurements were facilitated by the Inertial Measurement Unit (IMU) System Ultium Motion with the MyoResearch software suite, wherein data recording and synchronization were conducted (Ultium Motion and MR3.18, Noraxon U.S.A., Inc., Scottsdale, AZ, USA). The 7-IMU sensor setup (feet, shank, thigh, pelvis) and sensor calibration (reference pose, walking calibration) were facilitated, as recommended by the manufacturer [11]. Patients first acclimatized to the treadmill environment and their comfort speed (group: “self-paced velocity (SPV)”) was identified. The patients then walked at SPV for two minutes, followed by a 30-s data recording period. Next to an instrumented gait analysis as SPV, all patients were required to do a gait analysis at a speed of 2 km/h (group: “2 km/h”) and additionally at 4 km/h at 6- and 12-months follow-up (group: “4 km/h”).

### Data assessed and analyzed

**Spatio-temporal parameters:** Gait event detection and spatio-temporal parameters, including stance and swing phases, cadence, and stride length, were assessed via the pressure plate in the treadmill and analyzed using the corresponding software (MR3.18, Noraxon U.S.A., Inc., Scottsdale, AZ, USA).

**Kinematic parameters:** Kinematic parameters of the hip, knee, and ankle joints in the sagittal plane, and of the ankle joint in all three planes (sagittal, frontal, and transverse), were calculated according to the International Society of Biomechanics (ISB) standards using the MR3 software [12]. Kinematic data were further processed using MATLAB (The MathWorks Inc., Natick, MA, USA). They were segmented into gait cycles, normalized to 100%, and analyzed in increments of 1%.

**General parameters:** Furthermore, the general demographic, fracture details, complications were assessed.

**Handling of loss to follow-up and missing data:** Follow-up visits were scheduled at baseline, 6 weeks, 3 months, 6 months, and 12 months. Participants were considered lost to follow-up for a given visit if they did not attend the appointment or if the assessment occurred outside the prespecified visit window. In addition, data collection was stopped early for administrative reasons (April 1 st, 2025), resulting in administrative right-censoring for participants who had not yet reached later follow-up time points at the time of study termination.

For the primary longitudinal analyses, we performed a complete-case (per-protocol) analysis, including only participants with a complete gait-analysis dataset across all scheduled time points (i.e., all visits available within the defined follow-up windows). Participants with missing data at any time point were excluded from the longitudinal inferential analyses. No imputation of missing values was performed.

### Statistics

For the visual representation of the results, all patient data available at the respective time points were considered. Kinematic data were graphically represented as mean ± 1 standard deviation across the entire gait cycle. Spatio-temporal parameters were depicted using violin plots.

The analysis comprised of a longitudinal comparison (i.e. progression over one year) and a comparison to a healthy reference population (*n* = 30, Mean Age 27 ± 7 years, further information see [13]). The gait analysis setup was identical to the one described here (walking on a treadmill with 7 IMUs attached to the lower limbs). All healthy participants walked at a set speed of 4 km/h, which is why the patients’ data were compared with that of the healthy participants only at this speed.

Time-discrete spatio-temporal parameters were analyzed using repeated-measures ANOVA (*p* =.05), with Greenhouse-Geisser correction applied when sphericity was violated (Mauchly’s test). Effect sizes were reported as partial η² (≤ 0.01 small, ≥ 0.06 medium, ≥ 0.14 large). Post-hoc comparisons were performed using Bonferroni correction in SPSS (IBM Corp. Released 2024, IBM SPSS Statistics for MacOS, Version 30.0.0 Armonk, NY, USA).

Time-dependent kinematic data were analyzed using Statistical Parametric Mapping (SPM, spm1d.org [14]) and repeated-measures ANOVA. In cases of significant differences, paired t-tests with a Bonferroni-adjusted critical p-value (SPV and “2 km/h”: α = 0.05; number of tests = 4, critical p-value 0.0127; “4km/h”: α = 0.05; number of tests = 3, critical p-value 0.017) were conducted.

## Results

### Patient data

**Fig. 2:**
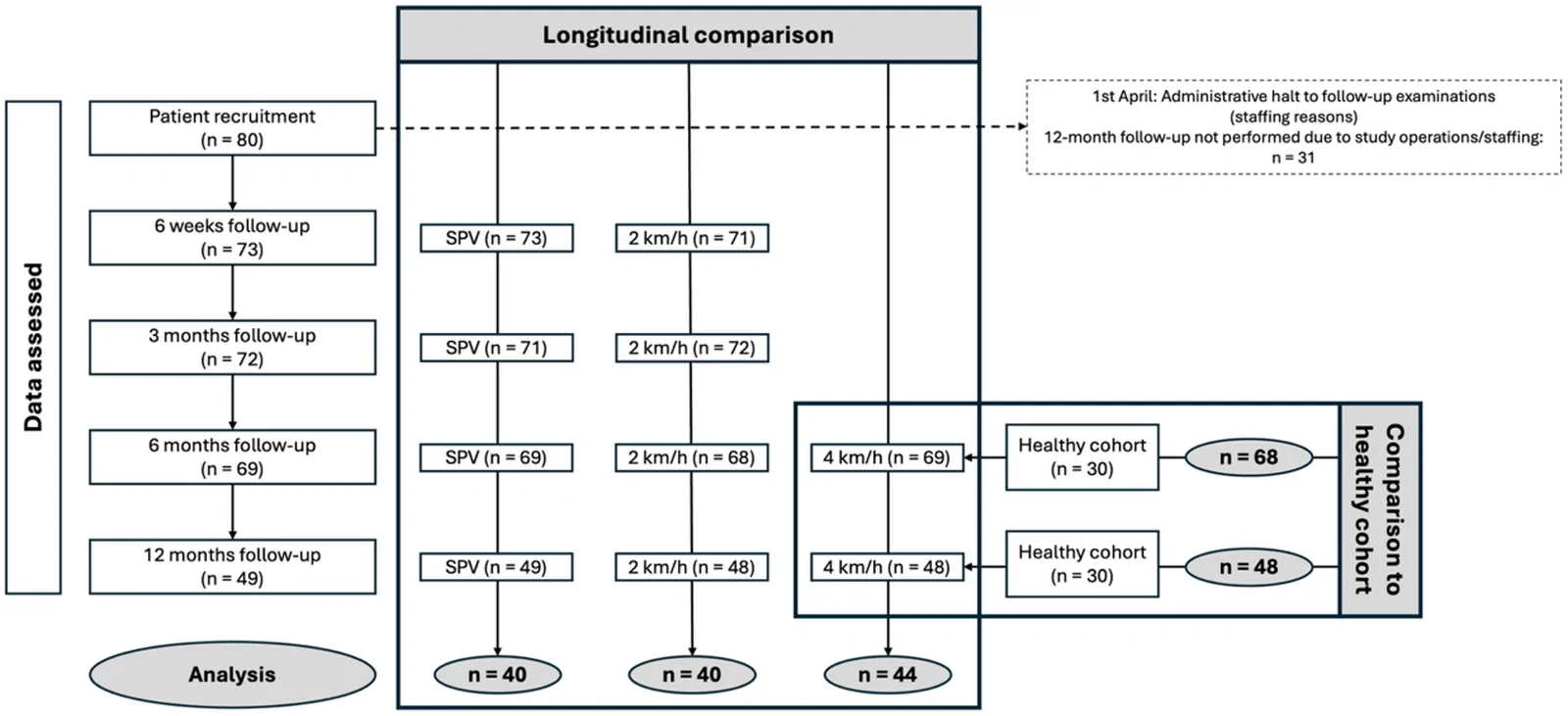
Flow chart of included patients and follow ups

A total of 80 patients were recruited (Mean age 41.1 ± 14.0; mean BMI of 25.3 ± 3.7; 41.3% female; Uni-/Bi-/Trimalleolar: 48%/28%/25%). During follow-up, some participants did not contribute data to specific visits because they did not attend appointments or because assessments fell outside the prespecified follow-up window. Furthermore, due to administrative reasons, gait-analysis data acquisition was terminated on April 1 st, 2025, leading to administrative censoring and fewer observations at later follow-up visits. The numbers of participants assessed at each follow-up visit and for each measurement condition, as well as the final complete-case sample used for longitudinal analyses, are shown in Fig. 2.

### Spatio-temporal parameters

**Fig. 3:**
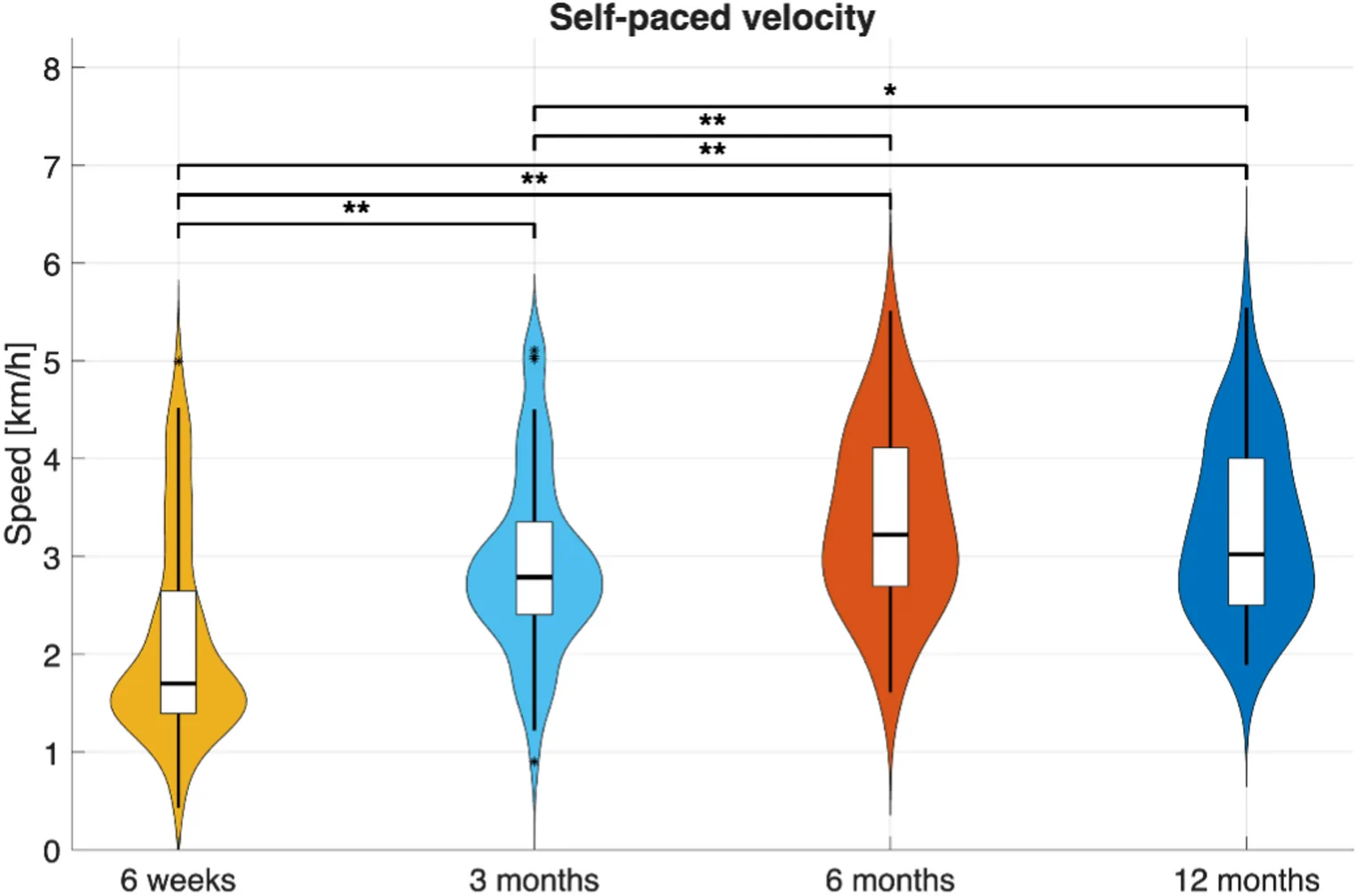
Self-paced walking velocity across follow-up time points; Significant differences between time points are marked with brackets; *p* <.05 (*) and *p* <.01 (**)

The mean speed at SPV increased significantly from 6 weeks (2.2 ± 1.1 km/h) to 3 months (2.9 ± 0.9 km/h), 6 months (3.4 ± 0.9 km/h), and 12 months (3.3 ± 0.9 km/h; F(1.850, 57.363) = 29.772, *p* <.001, partial η² = 0.490). Post-hoc comparisons revealed significant differences between 6 weeks and all later time points (all *p* <.003), and between 3 and 6 months (MD = 0.14 m/s, *p* =.002). No significant difference was observed between 6 and 12 months (*p* = 1.000) (Fig. 3).

**Fig. 4:**
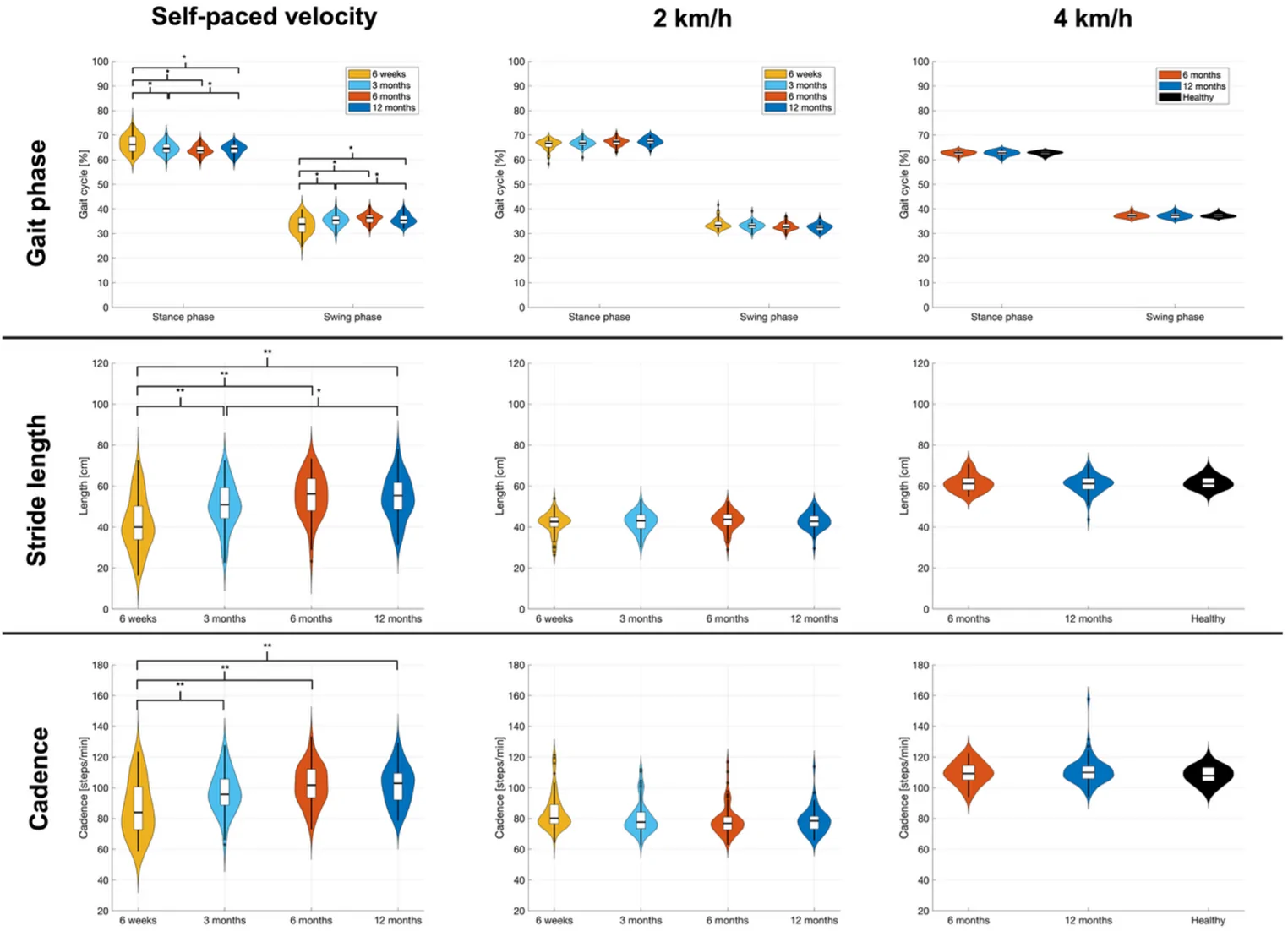
Comparison of spatio-temporal parameters for self-paced velocity, 2 km/h, and 4 km/h; significant differences between time points are marked with brackets; *p* <.05 (*) and *p* <.01 (**)

The remaining spatio-temporal parameters assessed are illustrated in Fig. 4. For the longitudinal assessment at SPV, all spatio-temporal parameters showed significant improvements over time (cadence (F(1.849, 73.956) = 26.503, *p* <.001, partial η² = 0.399), stance phase (F(1.908, 76.328) = 18.709, *p* <.001, η² = 0.319), swing phase (η² = 0.319, *p* <.001), and step length (F(1.831, 73.229) = 49.327, *p* <.001, η² = 0.552)). Post-hoc comparisons showed significant differences between 6 weeks and all later time points for all parameters (all *p* <.001). Changes between 3 and 6 months remained significant for cadence (*p* =.033), stance/swing phase (*p* =.019), and step length (*p* <.001), but not between 6 and 12 months (all *p* ≥.158). At fixed walking speeds (2 km/h or 4 km/h) no significant longitudinal differences were observed. For the comparison to the healthy reference group (4 km/h, between-subjects ANOVA), no significant differences were found at 6 or 12 months for any spatio-temporal parameter (all F < 1.6, *p* >.19, η² ≤ 0.021).

### Kinematic parameters

**Fig. 5:**
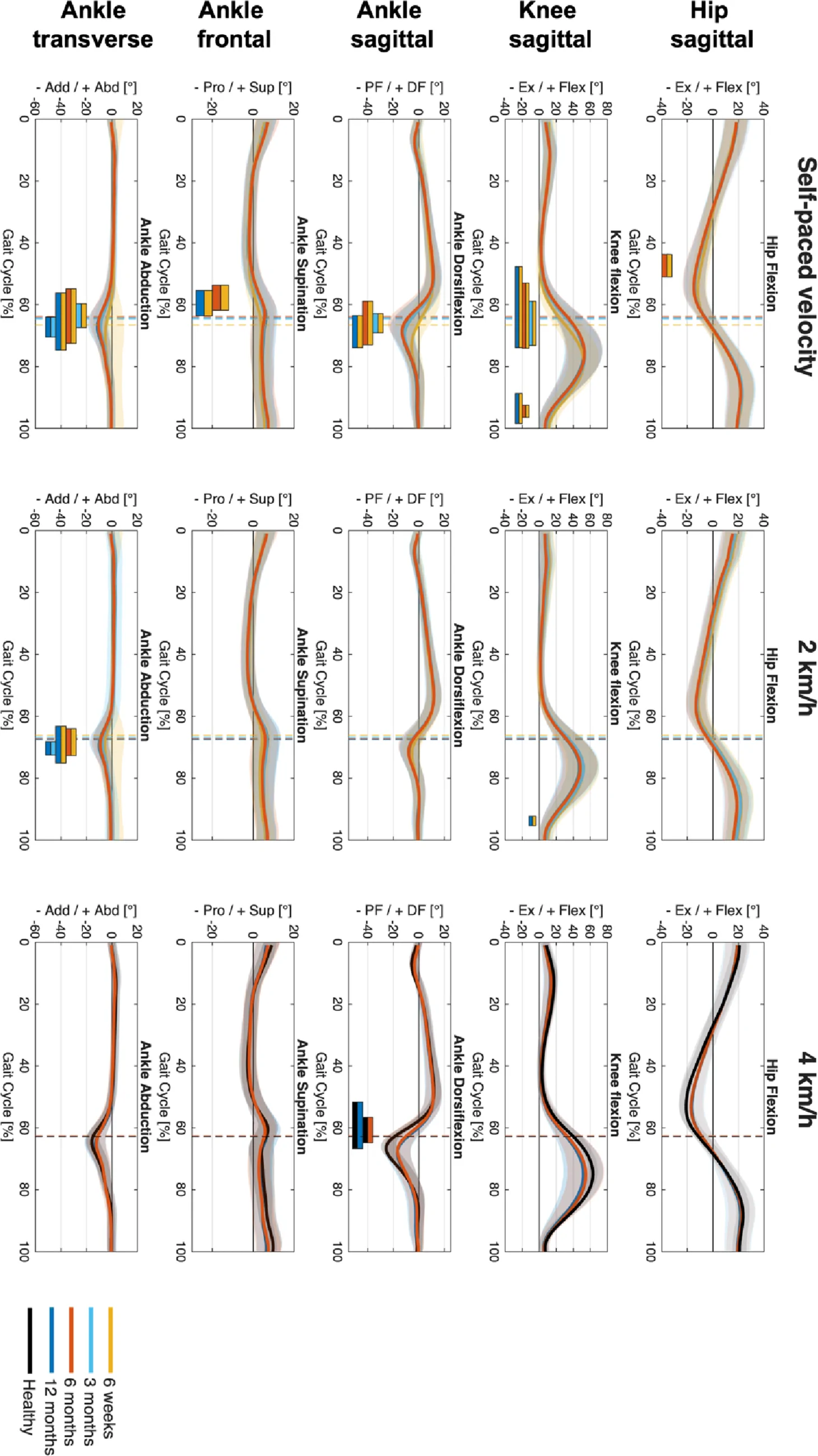
Kinematic parameters for hip (sagittal), knee (sagittal) and ankle (sagittal, frontal, transverse) for self-paced velocity, 2 km/h and 4 km/h at different timepoints (6 weeks, 3 months, 6 months, 12 months), with healthy controls shown for the 4 km/h condition; dashed line for toe-off

The kinematic analysis for hip (sagittal), knee (sagittal), and ankle (all three planes) are illustrated in Fig. 5. Significant longitudinal changes were predominantly seen at SPV for all three joints (hip, knee, ankle). At a speed of 2 km/h, significant changes were observed in ankle transverse kinematics and, in a smaller time window, knee sagittal kinematics. For the comparison to a healthy control the ankle joint (sagittal) was the only joint to show significant differences at 6 and 12 months at 4 km/h.

## Discussion

The herein presented study is the first to assess the longitudinal recovery at four time points of surgically treated ankle fractures and compare these results to a healthy control.

The study demonstrated a clear longitudinal, progressive gait recovery pattern. The most significant improvements occurred within the first 6 months. Gait analysis at SPV resulted in more significant results than both preset conditions “2 km/h” or “4 km/h”. The assessed spatio-temporal parameters showed greater differences than the kinematic parameters.

It is well established that walking speed influences kinematic parameters, including knee flexion, ankle plantarflexion, and hip motion. Consequently, kinematic changes observed at self-paced velocity (SPV) may partly reflect differences in walking speed rather than isolated biomechanical recovery. This is one reason why we additionally assessed patients at fixed speeds (2 km/h and 4 km/h): the comparatively limited number of significant longitudinal changes at fixed speeds supports that speed is a major contributor to SPV-driven changes. Importantly, the comparison of kinematic parameters to healthy controls was performed at 4 km/h rather than SPV, which substantially mitigates speed confounding for this comparison. Ideally, controls would have been individually speed-matched to each patient at SPV; the inability to do so represents a study limitation.

When compared to the healthy reference group at 4 km/h, the spatio-temporal parameters at 6 and 12 months showed no significant differences (all *p* >.19, η² ≤ 0.021), indicating normalization of temporal-spatial gait by 6 months. The kinematic parameters revealed persistent limitations at 6 and 12 months only for ankle sagittal measurements.

Only few studies have assessed longitudinal gait data following ankle fracture treatment. And these longitudinal data are usually limited to two time points [15, 16]. Most studies compare ankle fracture patients to a healthy control group [17–20]. Still, it seems reasonable that both approaches – longitudinal progression and comparison to a healthy control group – should be assessed, as they address different aspects of recovery. Longitudinal data allow the exploration of the gait recovery process. The comparison to a healthy control allows the identification of permanent deficits. To the best knowledge of the authors, the present study is the first to assess the longitudinal recovery of surgically treated ankle fractures at multiple time points with a comparison to a healthy control.

The analysis of longitudinal gait data facilitates the evaluation of recovery over time. Böpple et al. utilizing a marker-based motion capture system, reported a significant recovery in spatiotemporal parameters and gait kinematics between 2.3 and 6.5 months post-operatively (*n* = 14) [15]. Fernández-Gorgojo et al. assessed plantar pressure parameters in 22 patients at 6- and 12-months post-fracture [16]. They also observed an enhancement over time. The present study evaluated the recovery process at four distinct time points: 6 weeks, 3 months, 6 months, and 12 months. Compared to two time points, as done in previous studies, multiple time-point assessment allows for a better characterization of the recovery process. This allowed the authors, for the first time, to show, that the most significant progression occurs within the first six months. Substantial improvements were evident in both, the spatio-temporal and kinematic parameters.

The rehabilitation process is influenced by multiple factors, including age, injury severity, surgical treatment strategy, and the postoperative treatment regime. It can be assumed that the latter, i.e. the rehabilitation regime, is one of the most significant factors. Traditional postoperative regimes, as outlined in the introduction, comprise of immobilization and non- or partial weightbearing for a period of 6–8 weeks [3]. The herein conducted progressive rehabilitation regime, i.e. pain-dependent full weight bearing, possibly enhances the gait recovery process. This might be the reason, why the current study revealed the most significant improvements within the first 6 months, whereas the previously mentioned studies did show an improvement even after 6 months.

Whereas the longitudinal observation does allow to monitor the rehabilitation progress, the comparison to a healthy cohort allows to assess the degree of residual impairment or prove full recovery. Both above cited, longitudinal studies, also compared their final follow-up results to a healthy cohort and revealed residual impairments 6.5- and 12-months after ankle fracture [15]^,^ [16]. Tan et al. compared ankle fracture patients to a healthy cohort 4.5 years after ankle fracture surgery and was still able to show gait impairments [21]. The present study’s results align with a growing body of literature demonstrating that patients do improvement in gait parameters within the first year (longitudinal progression), they persistent with gait impairments compared to healthy controls after a year or more [17–20, 22]. These impairments affect various spatio-temporal parameters, including step/stride length, cadence, gait speed, stance/swing time, single support time, and an earlier foot-off time [8]. In the current study spatio-temporal parameters showed significant improvement in the longitudinal aspect mainly driven by an increase of the self-paced velocity. This is evidenced by the absence of any significant temporal variance in the observations conducted at a constant velocity. In the current study, by 12 months, hip and knee kinematics at 4 km/h approximated those of healthy controls, but significant deviations persisted in ankle sagittal parameters. Hoekstra et al. also reported a reduced range of motion in the affected ankle during key phases of gait at SPV [19]. Elbaz et al. on the contrary reported on kinematic alterations/compensations especially at the knee joint at SPV [22]. In the current study, alterations at the knee level were seen throughout the first 6 months for SPV, but not at 6- or 12-months follow-up compared to healthy controls, which was done at 4 km/h.

Ceiling effect in PROMs are well known. But similar effects might also be present in gait analysis. In the current study, the authors found the most pronounced differences at SPV. Predefined speeds, i.e. 2 km/h and 4 km/h often found no significant differences. One could postulate, that after a certain amount of time, patients have fully recovered at easy walking speeds. But gait is also influenced by the complexity of the tasks that needs to be performed. Walking at a slower speed over an even ground is most likely easier than walking over uneven grounds, running, or cutting exercises. More complex gait analysis, for example when running or performing certain cutting exercises, might be a promising approach to differentiate patients with a good outcome from those with a very good outcome. Moreover, it could help to identify persisting deficits that again could be a treatment target during rehabilitation.

This study had several limitations. First, the statistical analysis using ANOVA with SPM has not yet been fully validated. Notably, the current post hoc procedures available in the spm1d package are likely too simplistic, as they rely on a Bonferroni correction that assumes independence among post hoc tests – an assumption which does not typically hold for this type of data. As a result, the potential for type I or type II errors cannot be entirely excluded, and the interpretation of kinematic comparisons should be made with caution. Additionally, the use of Bonferroni correction for determining the critical p-value may have increased the risk of type II errors, possibly leading to an underestimation of significant effects. Another important limitation is the early termination of gait analysis in a subset of patients due to administrative constraints. This may have reduced the statistical power, particularly for detecting changes in the later stages of follow-up. Despite these limitations, the main trends observed – in particular, the early improvements followed by a plateau – offer valuable insights for clinical practice and the design of future studies. A further limitation is the age difference between the patient group (mean 41.1 ± 14.0 years) and the healthy control group (mean 27 ± 7 years). As gait characteristics change with age, this may have influenced the comparisons between groups. Age-matched reference data were not available; future studies should employ age-matched controls.

In contrast to many previous investigations, which have typically evaluated patients at a single follow-up time point, the present study contributes a comprehensive longitudinal perspective in a large cohort, delineating the trajectory of recovery across multiple intervals within the first year and thereby providing a more nuanced understanding of both the extent and the temporal dynamics of gait restoration after ankle fracture. These findings underscore the importance of ongoing objective gait assessment and targeted rehabilitation strategies to address persistent functional deficits and optimize long-term outcomes.

This persistent abnormality highlights a potential area for targeted intervention, as incomplete recovery at the ankle may contribute to ongoing functional limitations or compensatory strategies. Collectively, these findings underscore the importance of early, intensive rehabilitation and suggest that future interventions should address not only global walking ability but also joint-specific deficits – particularly at the ankle.

## Conclusion

The herein presented study is the first to assess the longitudinal rehabilitation at multiple time points of ankle fracture patients and compare these to a healthy control. Despite a progressive rehabilitation regime, patients do have impairments even after one year. Future studies should include more complex gait tasks, for example running or cutting exercises, to better understand the residual impairments.

## Data Availability

The data supporting the findings of this study are available from the corresponding author upon reasonable request.
