# Correction: Recovery of gait after ankle fracture surgery following early mobilization and weight-bearing

**DOI:** 10.1007/s00402-026-06513-y

**Published:** 2026-09-30

**Authors:** Leandra Bauer, Anne Sophie Schlegel, Anne Scheuer, Wolfgang Böcker, Hans Polzer, Sebastian Felix Baumbach

**Affiliations:** 1 Experimentel Orthopaedics, University Hospital Jena, Campus Eisenberg, Waldkliniken Eisenberg, Friedrich-Schiller-Universität Jena, Jena, Germany; 2 Department of Orthopaedics and Trauma Surgery, Musculoskeletal University Center Munich (MUM), University Hospital Munich, Ludwig-Maximilians-Universität München, Munich, Germany

**Correction: Archives of Orthopaedic and Trauma Surgery (2026) 146:289**

https://doi.org/10.1007/s00402-026-06472-4.

In this article, Fig. 5 incorrectly appeared as shown below,

**Figure:**
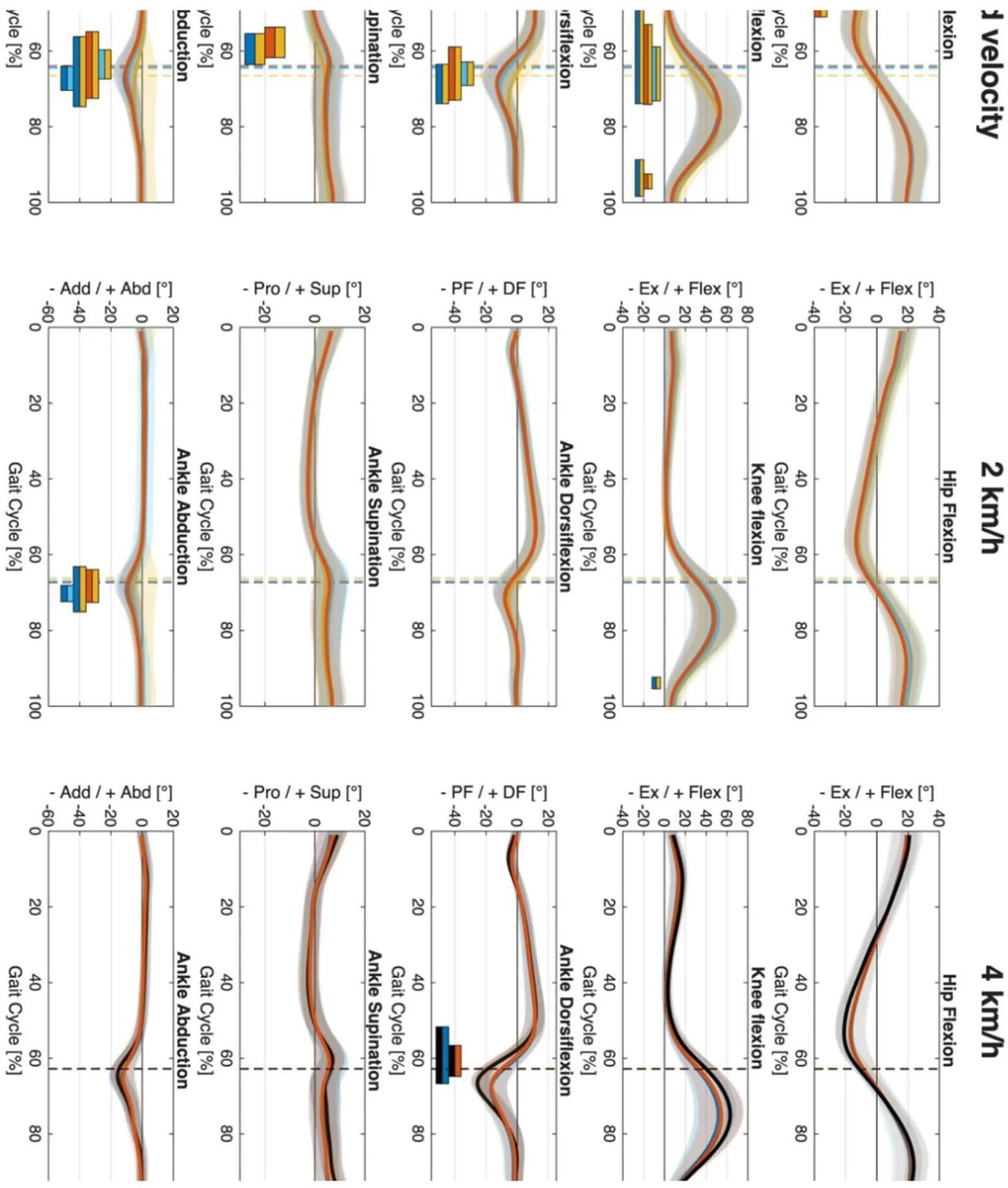

the figure(s) should have appeared as shown below

**Figure:**
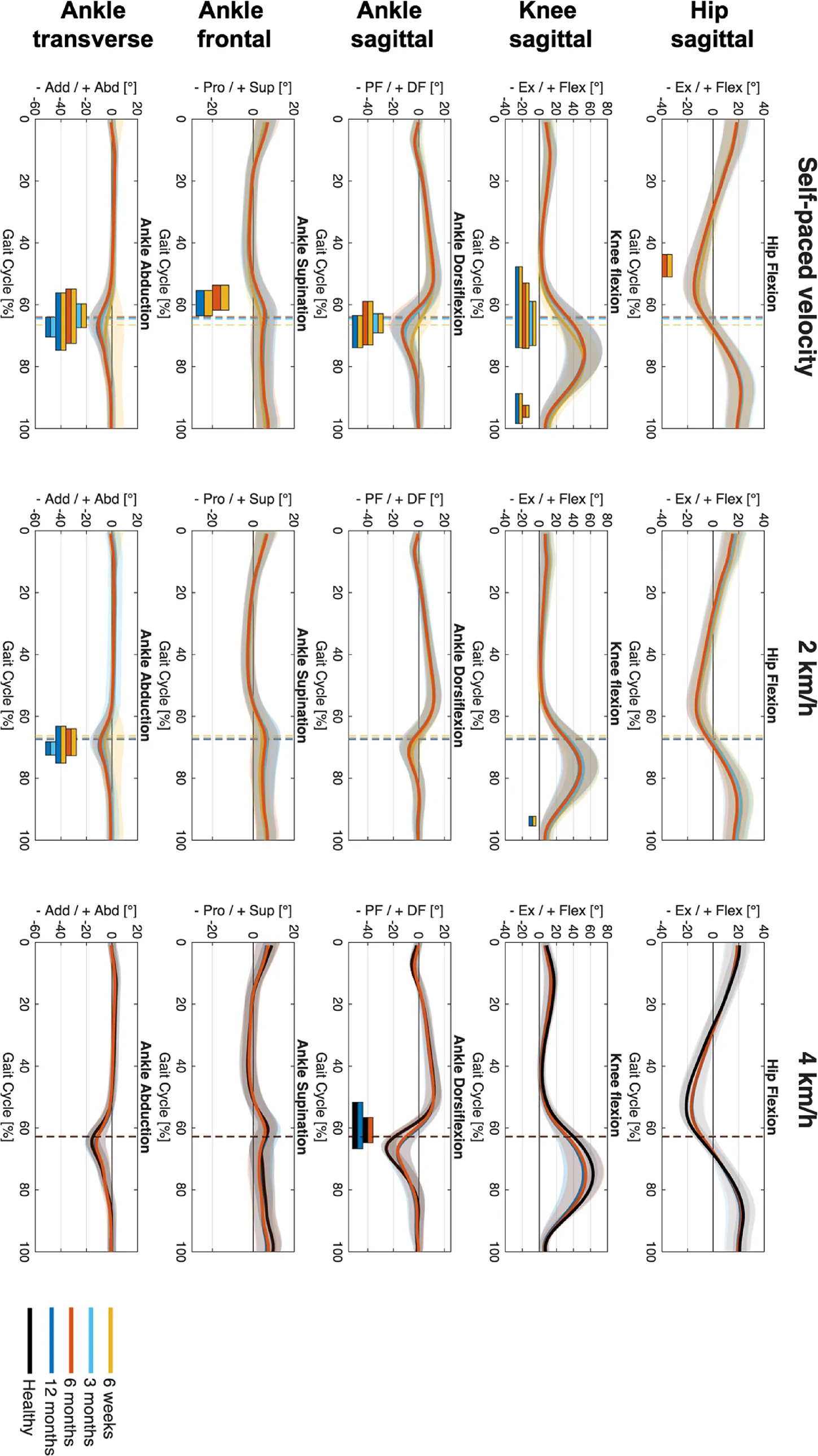

The original article has been corrected.

